# Stereotactic Re-Irradiation for Local Recurrence after Radical Prostatectomy and Radiation Therapy: A Retrospective Multicenter Study

**DOI:** 10.3390/cancers13174339

**Published:** 2021-08-27

**Authors:** Tanguy Perennec, Loig Vaugier, Alain Toledano, Nathaniel Scher, Astrid Thomin, Yoann Pointreau, Guillaume Janoray, Renaud De Crevoisier, Stéphane Supiot

**Affiliations:** 1 Department of Radiation Oncology, Institut de Cancérologie de l’Ouest, Boulevard J. Monod, 44800 St-Herblain, France; tanguy.perennec@ico.unicancer.fr (T.P.); Loig.vaugier@ico.unicancer.fr (L.V.); 2 Department of Radiation Oncology, Institut de Radiothérapie et Radiochirurgie Hartmann, 92300 Levallois, France; a.toledano@rt-hartmann.fr (A.T.); n.scher@rt-hartmann.fr (N.S.); 3 Department of Radiation Oncology, Bretonneau Hospital, 37000 Tours, France; a.thomin@chu-tours.fr (A.T.); guillaume.janoray@bordet.be (G.J.); 4 Department of Radiation Oncology, Centre Jean Bernard—Institut Inter-RégionaL de Cancérologie (ILC), 72000 Le Mans, France; y.pointreau@ilcgroupe.fr; 5 Department of Radiation Oncology, Institute Eugene Marquis, 35000 Rennes, France; r.de-crevoisier@rennes.unicancer.fr; 6 Centre de Recherche en Cancérologie Nantes-Angers (CRCNA), UMR 892 Inserm—6299 CNRS, Institut de Recherche en Santé de l’Université de Nantes, 8 Quai Moncousu, BP 70721, CEDEX 1, 44007 Nantes, France

**Keywords:** prostate cancer, stereotactic body radiation therapy, re-irradiation

## Abstract

**Simple Summary:**

Stereotactic body radiation therapy remains an understudied treatment option for local recurrence in the prostate bed after prostatectomy followed by radiation therapy. Ablative treatment of local recurrence could avoid or delay androgen deprivation therapy or next-generation antiandrogens. This study suggests that this treatment modality could be a valuable option if confirmed by a prospective study, but long-term toxicity may be a significant limitation.

**Abstract:**

Prostate cancer recurrence in patients previously treated with radical prostatectomy and radiation therapy is challenging. Re-irradiation could be an option, but data regarding efficacy and safety are lacking. We retrospectively evaluated salvage re-irradiation for local recurrence after prostatectomy and external beam radiation therapy. We collected data from 48 patients who underwent salvage reirradiation with stereotactic radiation therapy for local prostate cancer recurrence in the prostatic bed at four French centers. Fifteen patients (31%) were on androgen deprivation therapy during stereotactic radiotherapy. Biochemical response and relapse-free survival were analyzed, and post-treatment toxicities were assessed according to the Common Terminology of Adverse Events criteria. Five patients had grade 3 late bladder toxicity (cystitis), three had grade 3 late incontinence, and one had grade 3 late chronic pain. At three months, 83% of patients had a positive biochemical response. The median follow-up was 22 months. At the end of the follow-up, 21 patients (43%) had a biochemical relapse. The median time to biologic relapse was 27 months. The biochemical relapse rates at 1 and 2 years were 80% and 52%, respectively. In conclusion, salvage re-irradiation for recurrent prostate cancer in the prostate bed may generate significant toxicity rates, and a prospective study with appropriate patient selection is needed to evaluate its effectiveness.

## 1. Introduction

Prostate cancer is the third most frequently diagnosed and eighth-deadliest cancer worldwide, according to the Global Cancer Project (GLOBOCAN) report, which estimated 1,276,106 new cases and 358,989 deaths in 2018 [1]. Radical prostatectomy is widely offered for localized prostate cancer and is part of international guidelines [2,3]. A biochemical relapse, defined by a prostate-specific antigen (PSA) level above 0.2 ng/mL, occurs in 25–30% of patients treated by surgery within ten years [4,5,6]. Salvage radiation therapy (SRT) combined with androgen deprivation therapy (ADT) is then recommended if there is no evidence of distant metastatic disease [7]. Adjuvant radiation therapy (ART) immediately delivered after prostatectomy is also an option for high-risk diseases [8]. After salvage treatment, 20–38% of patients experience a new biochemical relapse within five years, depending on whether or not they are using hormone deprivation therapy [9]. ADT alone is recommended in case of additional recurrence, delayed if the risk of metastatic disease is low (PSA doubling time superior to 10 months), and associated with modern antiandrogen in the eventuality of a new progression [2,10,11]. ADT is known to have numerous side effects, such as sexual dysfunction, bone loss, metabolic changes, hot flushes, and increased cardiovascular events [12,13]. Stereotactic body radiation therapy (SBRT) is a radiotherapy technique that allows for high doses per fraction with a high dose gradient. This technique could be interesting at the first recurrence after radiation therapy or later at relapse under ADT. SBRT could preserve the adjacent organs and take advantage of the low alpha/beta ratio of prostate cancer [14,15]. It may also be a new curative therapeutic option to avoid or delay ADT, castration resistance, and pejorative events. We aimed to evaluate the safety and efficacy of the re-irradiation of a local recurrence in the prostatic bed.

## 2. Materials and Methods

### 2.1. Patients Selection and Data Collection

We retrospectively collected data from all patients treated with SBRT for local prostate cancer recurrence after radical prostatectomy and radiotherapy in four French radiotherapy departments in Nantes, Tours, Rennes, and Levallois. Data collection was based on medical records available at the time of data collection (during 2019). Patients were first selected based on the lists of patients treated on the dedicated stereotactic machines, filtered based on the diagnosis of prostate cancer. The maximum collection date initially set in the protocol was 2010, even though the installation date of the dedicated machines was later: 2011 in Tours and Nantes, and 2014 in Rennes and Levallois. Then, the patients initially selected had to meet the following inclusion criteria: localized prostate cancer treated by radical prostatectomy followed by radiotherapy (adjuvant or salvage), in strict local recurrence proven by MRI, choline PET-CT, or PSMA PET-CT. Patients had to be treated with stereotactic radiotherapy with ablative intent. We excluded patients with less than two months of follow-up at the time of data collection. Written consent was obtained from all patients to use their data for research purposes, and all patients were informed of the nature of the study and protocol by letter.

### 2.2. Treatment Modality

SBRT treatment was strictly limited to the area of recurrence, defined by a radiation oncologist using choline PET-CT or PSMA PET-CT, after fusion and recalibration, assisted by MRI, if performed. If the choline PET-CT and PSMA PET-CT had not been performed, delineation of the target volume was performed based on the MRI. There were no other specific rules for patient selection regarding treatment modalities, and therefore, additional treatment characteristics are presented in the results section.

### 2.3. Outcomes

Our primary endpoint was toxicity, evaluated according to the CTCAE v5.0 classification [16], using non-infectious cystitis criteria for bladder toxicity and proctitis criteria for rectal toxicity. Incontinence, erectile dysfunction, and abdominal pain were reported with the corresponding criteria. If toxicity was not explicitly noted in the medical record, the information was considered missing. Acute toxicity was defined as toxicity occurring within three months after the end of radiotherapy, while late toxicity was defined as toxicity occurring afterward. When a patient presented several symptoms of different grades in the same category, only the maximal grade was considered.

Treatment efficacy was the secondary endpoint. The initial response was defined as a relative decrease in PSA greater than 20%, three months after completion of SBRT. Biochemical relapse after SBRT was defined as an absolute increase in PSA greater than 0.2 ng/mL above the nadir.

### 2.4. Statistical Analysis

Statistical analysis was performed with R 3.6.1 (5 July 2019). The results for categorical variables are expressed as absolute numbers and percentages. The results for continuous variables are expressed as median (interquartile range). Available data are specified for each variable. Survival analysis was carried out by the Kaplan−Meier method with the Log-rank test (for univariate analysis) and Cox model, for which Schoenfeld residuals were tested to assess proportional hazards. The median follow-up time was calculated using the Kaplan−Meier estimator method with loss of follow-up treated as an event and death treated as a censored observation [17]. Multivariate Cox model analysis was performed using stepwise downward selection with Akaike information criterion (AIC) while maintaining well-known associated factors (time since irradiation, Gleason, and PSA) [18]. Tests associated with a *p*-value under 0.05 were considered statistically significant.

### 2.5. Review of the Literature

We included published cohort, case-control, and single-case studies available on PubMed on 20 June 2021. We combined the MeSH terms “prostate neoplasm” with “re-irradiation”, and “prostate neoplasm” with “radiosurgery” and the single term “salvage.” Our search revealed 25 and 34 articles, respectively, from which we selected studies that included one or more patients treated with SBRT on the prostate bed for local relapse after prostatectomy and EBRT. We excluded all studies that mixed results from another population (patients without prior prostatectomy or prior radiotherapy) if the patients meeting our inclusion criteria represented less than one-quarter of the total study population.

## 3. Results

### 3.1. Patients Baseline Characteristics

From September 2011 to December 2019, 48 patients were treated by SBRT for re-irradiation of local recurrence of prostate cancer in four French medical centers. All patients had been diagnosed with prostate cancer and underwent radical prostatectomy as their first treatment. Surgery was followed by adjuvant radiotherapy in eight patients (17%) and salvage radiotherapy in forty patients (83%). Twenty-eight patients were initially high risk (58%), fourteen patients were intermediate risk (29%), and three were low risk (6.1%), according to the D’Amico classification. The high proportion of high risk was partially due to T3a or T3b stages (52%). Patient characteristics at the time of surgery and first radiotherapy are summarized in Table 1.

### 3.2. SBRT Characteristics

Relapses in the prostate bed after ART or SRT were treated according to local practice. All patients had one relapse localized in the prostatic bed, from which 11 relapses were in the seminal vesicle bed (23%), three in contact with the rectum (6.3%), four with the bladder (8%), and one with vesicoureteral contact (2%). The dose prescribed and the fractionation of the radiotherapy was variable. The two most prescribed regimens were 30 Gy in five fractions (37% of patients) and 36 Gy in six fractions (33% of patients), with one rest day between each fraction (2 days for three patients). Twenty-nine patients (60%) were treated on a Cyberknife and 19 others on Linac. Daily CBCTs were performed for all patients treated on Linac, except for two patients with fiducial placement. During re-irradiation, 15 patients were treated with ADT (31%), of which six started ADT at relapse. The remaining nine patients had started ADT more than three months before SBRT, ranging from 29 to 110 months (median, 57 months). As the relapse occurred while they were under ADT, those patients were non-metastatic castration-resistant patients. One patient received second-line hormone therapy (enzalutamide) before SBRT. The modalities and characteristics of SBRT are summarized in Table 2.

### 3.3. Initial Response

**Figure 1:**
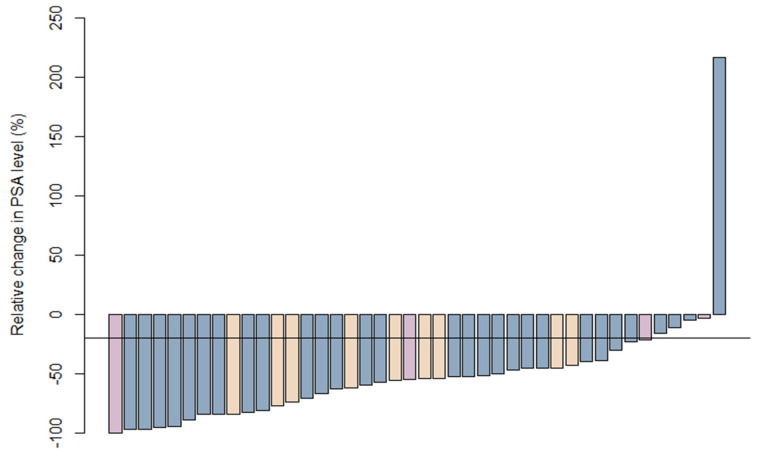
Relative PSA decline at three months after SBRT. Patients with a new ADT prescription are purple, and patients with ADT for more than three months are yellow. The horizontal line stands for −20% in the change of PSA. SBRT—stereotactic body radiation therapy; PSA—prostate specific antigen.

An initial response occurred in 37 of 42 patients (88%). The median relative change in PSA was −55% (range −100% to 217%). Five patients had no initial response, one of which had an increase in PSA (Figure 1), with lymph node relapse characterized by choline PET-CT. Of the remaining four, three relapsed in the prostatic bed, but only later (27, 31, and 38 months). All of the patients with no initial response had undergone pelvic MRI and choline PET-CT before SBRT, demonstrating local relapse only in the prostate bed. In these patients, however, no PSMA-PET had been performed.

### 3.4. Safety

Treatment was well tolerated during irradiation, as no grade 2 acute toxicity or higher was reported.

However, eight patients experienced late grade 3 toxicity, including five patients with late grade 3 bladder toxicity, three late grade 3 incontinence toxicities, and one chronic grade 3 abdominal pain. One patient experienced both grade 3 incontinence and cystitis. Erectile dysfunction was not detailed due to a large amount of unreported information. We report all toxicities in Table 3.

Among the five patients with late grade 3 cystitis, two patients had chronic cystitis after the first radiotherapy (grade 1). Regarding incontinence late toxicity, one patient already had grade 3 incontinence before SBRT, and one had grade 1. None of the patients with late cystitis or late urinary incontinence had bladder contact with the target volume. The dose prescription and toxicity after the first radiation therapy of these patients is summarized in Table 4.

The patient with grade 3 abdominal pain was treated with 35 grays in five fractions, with concomitant ADT. The target volume location was the vesicourethral anastomosis.

### 3.5. Survival Analysis

**Figure 2:**
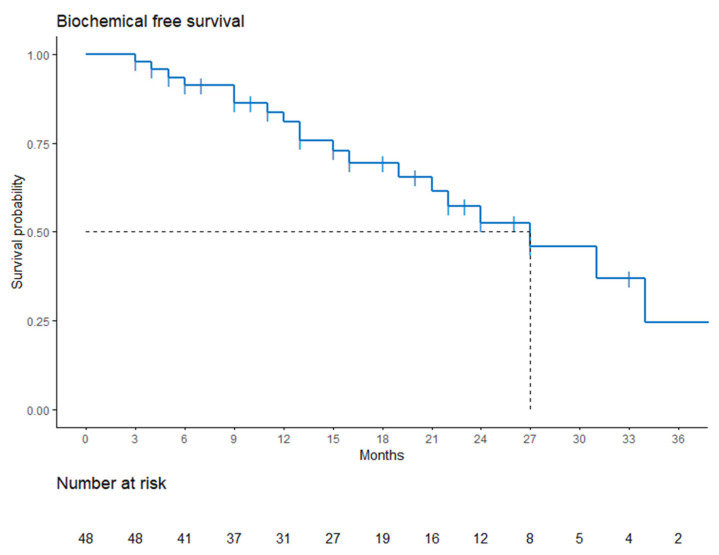
Biochemical free survival.

At the time of our analysis, the estimated median follow-up was 22 months. Twenty-one patients (44%) relapsed, from which thirteen patients had evidence of a prostatic bed relapse, with nine in the re-irradiated territory. Two patients had a diagnosis of metastases (among which one also had a prostatic bed relapse) at relapse. Finally, four patients had a biochemical relapse with no evidence of local relapse nor metastases, even though next-generation imaging (Choline PET or PSMA PET) had only been performed in two of the four patients. One patient died during follow-up due to severe hepatic insufficiency secondary to liver metastases. Median biochemical recurrence-free survival was 27 months. The 1- and 2-year probability survival rates were 80% and 52%, respectively. Kaplan−Meier biochemical recurrence-free survival (BRFS) estimate is available in Figure 2.

**Figure 3:**
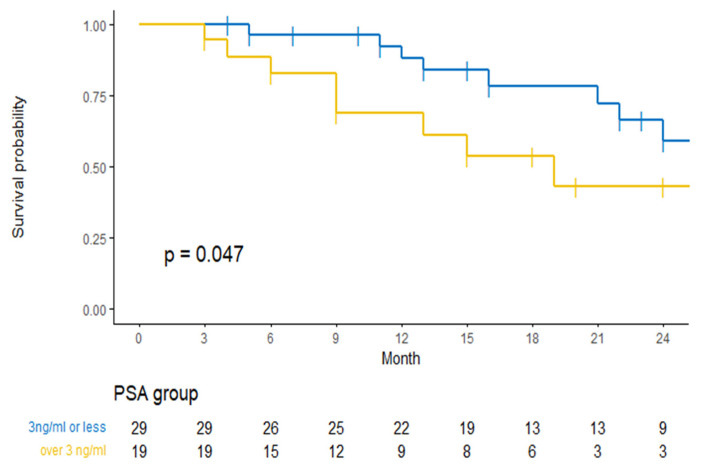
Biochemical relapse-free survival according to the pre-SBRT PSA level. SBRT-stereotactic body radiation therapy; PSA-prostate specific antigen.

Multivariable Cox regression analysis for BFRS failed to find statistically significant predictors of biochemical free survival (Table 5). In a univariable analysis, an increase in the PSA level before SBRT (HR = 1.19 (1.01; 1.41) *p* = 0.04) was slightly associated with a poorer BRFS. The difference between a high PSA before SBRT and a lower one (cut-off: 3 ng/mL) is illustrated by the Kaplan−Meier method in Figure 3.

## 4. Discussion

Treatment of local relapse after prostatectomy and radiation therapy is a difficult issue. Even if no evidence of metastatic disease is diagnosed, local ablative therapies such as SBRT are not the norm. In our study, SBRT resulted in a biochemical recurrence-free survival of 27 months and a biochemical recurrence-free survival rate of 80% at one year and 52% at two years, which is consistent with previous retrospective studies [19,20,21,22,23,24,25] (Table 6). Late urinary toxicity was significant and greater than in the literature, making it the major limitation to its use, pending more accurate information from prospective trials.

Assessment of efficacy is made difficult by the lack of consensus on biochemical relapse criterion for patients treated with prostatectomy and radiotherapy. Therefore, it is not easy to choose one for our study. As the patients had all undergone prostate surgery, it might be logical to choose a PSA level above 0.2 ng/mL as the criterion, like after the prostatectomy. However, none of the articles in the literature review chose this criterion. Olivier et al. chose a PSA level greater than nadir + 0.2 ng/mL for two samples [19]; Zerini et al. [21] preferred an absolute increase for two samples; and Volpe et al. [22] used the Phoenix criterion, i.e., a PSA increase greater than nadir + 2 ng/mL (which may have been guided by the presence of patients with prostate in place). Detti et al., D’Agostino et al., and Loi et al. [20,23,24] did not specify the criterion used. Other articles analyzing relapses after surgery and radiotherapy may have chosen another criterion, such as a PSA elevation greater than 0.5 ng/mL [7]. The criterion based on a PSA level above 0.2 ng/mL alone may be problematic because it is not uncommon for PSA levels to decline only gradually. For example, we had only 10 patients out of 48 (20%) who reached the 0.2 ng/mL threshold at six months. Of these ten patients, four did not reach this criterion at three months. Seven patients who had not reached this criterion at three months (PSA between 0.21 and 0.77) finally reached it between 6 and 12 months after the end of SBRT. We chose a criterion based on nadir so as to be comparable to other studies. We chose 0.2 ng/mL rather than 0.5 or 2 ng/mL above nadir, similar to other studies, because it was the most restrictive criterion.

Given the number of biochemical relapses at the end of follow-up (43%), SBRT could be proposed as an option to delay ADT, but not as a definitive treatment, unless criteria are defined to identify the best candidates. We were unable to identify clear predictors of recurrence-free survival. A high PSA level before SBRT appears to be associated with poor outcomes, but we lack the power to prove it or to identify other co-factors.

Nevertheless, treatment efficacy is not properly assessable here because of many confounding factors, such as the dose of SBRT or the prescription of ADT, as well as the small number of patients and their heterogeneity. For instance, some of the patients in this study were already under ADT before considering SBRT and were only offered SBRT at relapse, when they were castration resistant. Even though we took this into account in the multivariate Cox model, the objectives were not identical in those populations. We considered a delay in the onset of ADT and a sufficient biochemical survival rate as good objectives for hormone naive patients, but this cannot be used with more advanced diseases already on ADT, for which specific mortality or metastasis-free survival might be more appropriate. Unfortunately, we have no sufficient follow-up to analyze these elements. The heterogeneity of next-generation imaging prescriptions (PSMA PET-CT and choline PET-CT) is another limitation for determining the best candidates for this treatment modality, given the greater sensitivity and specificity of PSMA PET-CT compared with choline PET-CT [26,27,28]. We could not compare the results using imaging modality because of the small number of patients and the many confounding factors, such as ADT prescription, that can influence the imaging results. PSMA PET-CT sometimes suffers from its availability, but given the importance of proper patient selection because of potential toxicities, it seems necessary to consider always performing it before such treatment.

In addition, some data on the first treatments (prostatectomy and radiotherapy) are missing, such as the dose or time to SBRT, which may interfere with the interpretation of the results. This is because some patients were treated several years before (eight patients had their first radiotherapy before 2004), sometimes in another center than the one performing SBRT where the data were collected. Indeed, only a few expert centers practice this technique and many patients were referred from other centers.

A randomized controlled trial comparing SBRT to observation or versus next-generation antiandrogen for recurrent patients already treated by ADT would provide a definitive answer on its place in patient management. Similarly, an update of this study with more hindsight would help to appreciate its long-term utility better. It is therefore reasonable to wait for a prospective study controlling these biases in order to know its actual effectiveness. This therapeutic option could also be compared, or at least discussed, with other local salvage treatments. For instance, one study assessed the feasibility of salvage surgery, with a comparable median of biochemical recurrence-free survival of 23.7 months [29]. Another study reported the feasibility of brachytherapy with five patients, which is not enough to make conclusions on efficacy and safety [30].

The toxicity of this treatment is substantial, especially as the average follow-up is relatively low (22 months), and not all potential toxicities could be observed. Unfortunately, the small number of patients and the heterogeneity of dose prescription did not allow us to perform a statistical analysis with sufficient power to conclude. Nevertheless, it is important to note that the irradiations that resulted in late urinary grade 3 toxicity were not in contact with the bladder. Even if these results are only preliminary and do not allow us to establish a causal link or to identify contributing factors, these results nevertheless encourage us to be very cautious and to select patients carefully.

## 5. Conclusions

Stereotactic body radiation therapy for local relapse can provide a 2-year biochemical rate-free survival over 50% at the cost of late toxicities with some grade 3. This study demonstrates the interest in this technique, but requires further investigations by prospective trials to be usable in current practice. In any case, the risks of long-term toxicity, especially urinary toxicity, must encourage great prudence, careful selection, and detailed information of patients.

## Figures and Tables

**Table 1:** Patient initial characteristics.

Characteristics	Available Data	Overall
Age at diagnosis	48	61 (48–75)
D’Amico initial risk	45	
Low		3 (6.2%)
Intermediate		14 (29.2%)
High		28 (58.3%)
Gleason score	46	
6		12 (25%)
7		33 (68.8%)
8		1 (2.1%)
ISUP grade	43	
ISUP 1		12 (25%)
ISUP 2		19 (39.6%)
ISUP 3		11 (22.9%)
ISUP 4		1 (2.1%)
PSA (ng/mL)	41	9 (4.8–38)
pT3a or above	42	25 (52.1%)
pN1	45	6 (12.5%)
Positive margins	47	9 (18.8%)
Time to irradiation (month)	47	26 (2–108)
RT indication	48	
Adjuvant		8 (16.7%)
Salvage		40 (82.3%)
Dose delivered to the prostatic bed (Gy)	41	66 (60–75)
Pelvic node irradiation	48	5 (10.4%)
ADT associated with irradiation	48	4 (8.3%)

ADT—androgen deprivation therapy; Gy—gray; ISUP grade—International Society of Urological Pathology grade.

**Table 2:** Characteristics at re-irradiation.

Characteristics	Available Data	Overall
Delay since first irradiation (month)	47	102 (33–210)
PSA prior to SBRT (ng/mL)	48	2.6 (0.2–10.4)
ADT during SBRT	48	15 (31.2%)
Among which…		
Long term ADT (>3 months before SBRT)		9 (18.8%)
ADT beginning along the SBRT		6 (12.5%)
Exams before SBRT	47	
Choline PET-CT alone		11 (23%)
Choline PET-CT + MRI		28 (59%)
Choline PET-CT + PSMA PET/CT		4 (8.5%)
Choline PET-CT + PSMA PET/CT + MRI		2 (4.2%)
PSMA PET-CT + MRI		1 (2.1%)
MRI + CT scan + bone scintigraphy		1 (2.1%)
Total dose (Gy)	48	31.5 (20–37.2)
Fractionation (days)	48	5 (3–6)
SBRT course	48	
30 Gy in 5 fractions		18 (37.5%)
36 Gy in 6 fractions		16 (33.3%)
Other		13 (27.1%)

ADT—androgen deprivation therapy; Gy—gray; SBRT—stereotactic body radiation therapy.

**Table 3:** Acute and late toxicity associated with stereotactic body radiation therapy.

	Available Data	Grade 1	Grade 2	Grade 3
Acute rectal toxicity	47	2 (4.3%)	1 (2.1%)	-
Acute bladder toxicity	47	5 (10.6%)	2 (4.3%)	-
Late proctitis	44	4 (9.1%)	3 (6.8%)	-
Late cystitis	44	8 (18.2%)	4 (9.1%)	5 (11.4%)
Late urinary incontinence	45	7 (15.6%)	3 (6.7%)	3 (6.7%)
Chronic abdominal pain	44	3 (6.8%)	-	1 (2.3%)

**Table 4:** Main characteristics of the patients with a grade 3 urinary toxicity.

Late Toxicity after SBRT		SBRT Prescription	ADT during SBRT	Time Since First Radiotherapy (Months)	Late Toxicity Post First Radiotherapy	
Incontinence	Cystitis				Incontinence	Cystitis
0	3	37.25 Gy in 5	no	75	1	1
3	0	35 Gy in 5	no	95	3	0
0	3	36 Gy in 6	no	158	0	0
3	-	36 Gy in 6	no	126	1	1
3	3	30 Gy in 5	yes	-	0	0
0	3	36 Gy in 6	no	135	1	1
0	3	36 Gy in 6	yes	76	0	0

SBRT—stereotactic body radiation therapy; PSA—prostate specific antigen; ADT—androgen deprivation therapy; Gy—gray.

**Table 5:** Cox regression for biochemical free survival.

	Univariate Model			Multivariate Model		
	HR	IC95%	*p*-value	HR	IC95%	*p*-Value
Time since first RT	1.01	[1.00; 1.02]	0.16	1.00	[0.99;1.02]	0.58
PSA level before SBRT	1.19	[1.01; 1.41]	0.04	1.18	[0.96;1.45]	0.12
No ADT (reference)	1	-	-	-	-	-
ADT over 3 months before SBRT	2.03	[0.63; 6.58]	0.24	-	-	-
ADT starting at SBRT or less than three months before	0.91	[0.25; 3.38]	0.89	-	-	-
SBRT course:						
- 30 Gy in 5 fractions	0.57	[0.20; 1.68]	0.31	-	-	-
- 36 Gy in 6 fractions	0.33	[0.09; 1.25]	0.10	-	-	-
- others (reference)	1	-	-	-	-	-
Initial Gleason						
- 6 (reference)	1	-	-	1	-	-
- 7	0.70	[0.26; 1.9]	0.48	1.05	[0.36; 3.08]	0.93
- 8	1.08	[0.13; 9.36]	0.94	2.40	[0.24; 24.87]	0.46

SBRT—stereotactic body radiation therapy; PSA—prostate specific antigen; ADT—androgen deprivation therapy.

**Table 6:** Re-irradiation of prostatic bed: summary of the literature.

Ref.	Patients	Dose	ADT	BR	1-Year BRFS	2-Years BRFS	Grade 3 Acute Toxicity	Grade 3 Late Toxicity
Olivier et al. [19]	12	36 Gy in 6	2 (17%)	10 (83%)	0.79	0.56	0	0
Detti et al. [20]	8	30 Gy in 5	1 (12%)	7 (88%)	0.62	-	0	0
Zerini et al. [21]	10	25–30 Gy in 5–10	3 (30%)	-	40% *	-	0	0
Volpe et al. [22]	2	25–30 Gy in 5–6	0	2 (100%)	1	1	0	0
D’Agostino et al. [23]	8	25–30 Gy in 5	0	8 (100%)	81.6% *	41.7% *	1 hematuria	1 urethral obstruction
Loi et al. [24]	22	30 Gy in 5	17 (78%)	19 (86%)	80% *	-	1 urine retention	1 hematuria
Arcangeli et al. [25]	1	30 Gy in 5	1 (100%)	1 (100%)	-	-	0	-

BR—biochemical response; BRFS—biochemical relapse-free survival; ADT—androgen deprivation therapy. * Results from prostate and prostatic bed re-irradiation are mixed.

## Data Availability

The data presented in this study are available upon request from the corresponding author. The data are not publicly available for confidentiality reasons.
